# The Influence of a Working Memory Task on Affective Perception of Facial Expressions

**DOI:** 10.1371/journal.pone.0111074

**Published:** 2014-10-27

**Authors:** Seung-Lark Lim, Amanda S. Bruce, Robin L. Aupperle

**Affiliations:** 1 Department of Psychology, University of Missouri Kansas City, Kansas City, Missouri, United States of America; 2 Department of Pediatrics, University of Kansas Medical Center, Kansas City, Kansas, United States of America; 3 Center for Children's Healthy Lifestyle and Nutrition, Children's Mercy Hospital, Kansas City, Missouri, United States of America; The University of New South Wales, Australia

## Abstract

In a dual-task paradigm, participants performed a spatial location working memory task and a forced two-choice perceptual decision task (neutral vs. fearful) with gradually morphed emotional faces (neutral ∼ fearful). Task-irrelevant word distractors (negative, neutral, and control) were experimentally manipulated during spatial working memory encoding. We hypothesized that, if affective perception is influenced by concurrent cognitive load using a working memory task, task-irrelevant emotional distractors would bias subsequent perceptual decision-making on ambiguous facial expression. We found that when either neutral or negative emotional words were presented as task-irrelevant working-memory distractors, participants more frequently reported fearful face perception - but only at the higher emotional intensity levels of morphed faces. Also, the affective perception bias due to negative emotional distractors correlated with a decrease in working memory performance. Taken together, our findings suggest that concurrent working memory load by task-irrelevant distractors has an impact on affective perception of facial expressions.

## Introduction

Facial expression perception is known to be universal across different cultures to a certain extent [1], [2], but not entirely [3]. Affective perception of facial expressions is not solely determined by the physical configuration or properties of faces. In our daily life, face perception typically occurs in social contexts rather than as an isolated event. Indeed, affective perception is a highly subjective experience and it is significantly influenced by the situational context as well as perceiver-related individual differences [4].

The situational context impacts how facial expressions are perceived and decoded. Multisensory information (visual and/or auditory) or languages (verbal and/or nonverbal) simultaneously presented with facial expressions can substantially influence the perceptual and neural processing of faces [5]–[13]. Typically, the affective context increases emotional responses in valence congruent ways, and the contextual effect becomes more powerful when facial expressions are ambiguous. For example, emotionally ambiguous faces (50% fearful) were perceived as more fearful when threatening surrounding images were concurrently presented, while emotionally ambiguous faces (50% fearful) were perceived as less fearful when positive surrounding images were currently presented [8]. This contextual effect has been observed even when participants were instructed to make their decision exclusively based on facial stimuli while disregarding the contextual information, suggesting the automaticity of emotional face-context integration [14], [15]. This emphasizes that the contextual features surrounding the faces can have a critical and inevitable role on facial emotion perception.

Besides these concurrent external features, perceiver-related variables also dramatically affect the perception of facial expression. To make adaptive behavioral decisions in a given situation, the perceived face is often compared to faces previously encountered and encoded in our memory system. Thus, the perceiver's previous experience and knowledge, which includes both implicit and explicit affective-social learning and attitudes, can also substantially shape the perceptual and neural processing of faces [12], [16]–[21]. Not surprisingly, the personality traits of perceivers also significantly influence the perception of facial expressions [22]. Individual differences in anxiety and the behavioral activation and inhibition system have been frequently implicated in the perception and neural processing of facial expressions [8], [23], [24].

While many studies have focused on how emotional context and personality traits modulate the perception of facial expressions, relatively little is known about the cognitive components involved in perceiving facial expressions. In the past two decades, research shows that affective perception is not purely independent from the cognitive process of a perceiver [25], [26]. The perceptual decision-making process of facial expressions indeed requires and interacts with cognitive resources [16], [27], [28]. Facial emotion categorization often involves higher-level cognitive processes that include retrieving sematic labels (e.g., fearful) and their associated features from memory and evaluating perceived physical features of faces in relation to the retrieved internal concepts, which depends on fronto-temporal-parietal brain networks [29]–[31]. In particular, executive functioning has been proposed as a critical cognitive operation that dynamically orchestrates this cognitive-emotional interaction [25], [26]. Deficits in working memory, a core executive function, are common among patient populations that also show difficulties in social interactions [32]–[34]. Further, it has been shown that social-emotional reasoning, as measured by theory of mind and deontic selection tasks, is significantly impaired as a function of cognitive demands required by working memory tasks in a dual task paradigm. [35]. Taken together, the previous research suggests that affective and cognitive processes are directly or indirectly affected by shared processing resources or their reciprocal relationship [25], [26], [36], [37]. However, it is yet unknown whether concurrent working memory recruitment biases perceptual decision-making of facial expressions.

How does a demanding working memory task influence affective perception? Can task-irrelevant emotional distractors that influence ongoing executive function systematically bias subsequent affective perception on ambiguous emotional faces? To examine these questions, we asked participants to complete a dual-task paradigm involving both a spatial location working memory task and a forced two-choice perceptual decision task (neutral vs. fearful) with gradually morphed emotional faces (neutral ∼ fearful). Task-irrelevant word distractors (negative, neutral and control) were experimentally manipulated at the time of spatial working memory encoding. We hypothesized that, if affective perception is influenced by ongoing working memory-related processes, task-irrelevant emotional distractors occupied in the working memory system would bias subsequent perceptual decision-making on facial expression. Specifically, we tested two research hypotheses. First, if affective perception is influenced by working memory, task-irrelevant distractors (regardless of the emotional content) would bias perceptual judgments of facial expression. Further, we hypothesized that emotional distractors would have more of an influence on both working memory performance and subsequent judgments of facial expression as compared to neutral distractors.

These hypotheses were tested in part by examining whether responses were most consistent with the contrast gain model, which predicts that changes in perception occur when the intensity of the stimulus is intermediate, versus the response gain model, which predicts that changes in perception are proportional to the intensity of the stimulus and thus become evident at the high levels of emotional intensity perception [38]–[41] (see methods for additional information about these models). The current study advances previous research by (a) clarifying how working memory and emotional processing interact to influence one another and (b) examining whether the response gain or contrast gain model best explains the effect of cognitive-affective working memory manipulation on perceptual decision-making of facial expressions.

## Methods

### Participants

Thirty-eight healthy adult participants (Mean 30.5 years old ± SD 8.5; 24 males) were recruited for the experiment. All participants had normal or corrected-to-normal vision. The study protocol was reviewed and approved by the Institutional Review Board of the University of Missouri – Kansas City. Participants provided their written informed consent and completed the State-Trait Anxiety Inventory [42] and the BIS/BAS scale [43] prior to the experiment. Five participants who did not complete the self-report measures were not included in the correlational analysis.

### Stimulus Materials

**Figure 1:**
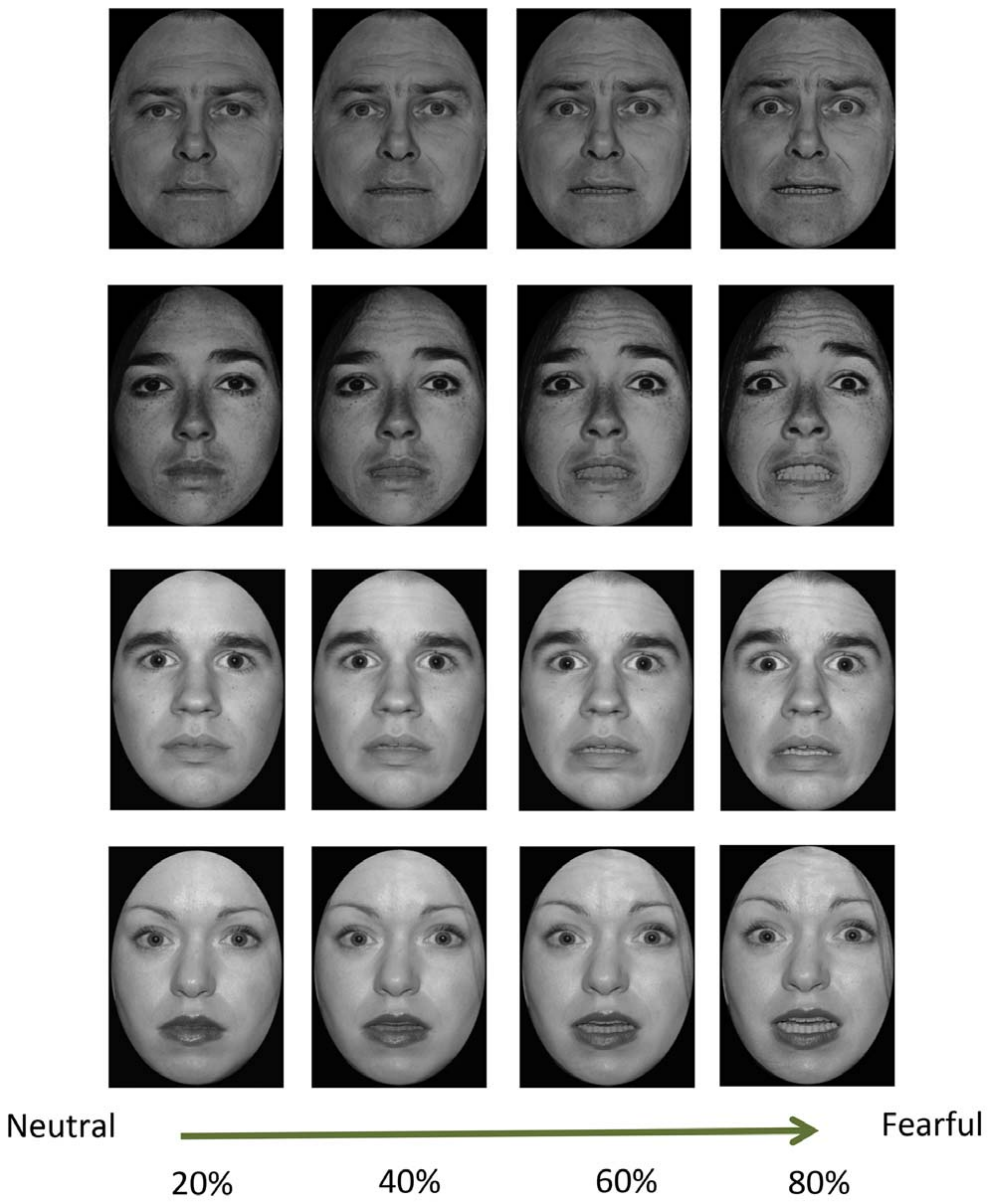
Experimental stimuli used for the affective perceptual decision task. Faces ranged from neutral to fearful in 20% increments. Facial stimuli were selected from Ekman set (Ekman & Priesen, 1976) and KDEF set (Lundqvist et al., 1988).

Emotional face stimuli (6 identities; 3 males and 3 females) were taken from the Karolinska Directed Emotional Faces (KDEF) set [44] and the Ekman face set [39], [45]. Most of the hair and non-facial contours were removed from the original images. To parametrically vary emotional expression, faces were morphed from neutral (0%) to fearful (100%) in 20% increments for each identity. Graphical morphing of facial stimuli was done by using the FantaMorph software (Abrosoft, Beijing, China). For the face task, 20%, 40%, 60% and 80% morphed faces were used (See Figure 1 for examples). Two identities (1 male & 1 female from KDEF set) were used for practice trials and four identities (2 males & 2 females from KDEF and Ekman face sets) were used for the main task trials. All face images (184×278 pixels) were shown in black and white. Negative and Neutral word stimuli for the working memory task were selected from a word set used in previous research [46] (see ref. for full stimulus list) - the negative category set contained 24 words judged to be emotionally threatening and the neutral category set contained 24 words judged to be neutral in this previous study; the stimulus word sets were matched for word frequency in the English language. For practice trials, separate word sets (12 negative words and 12 neutral words) were used.

### Procedure

**Figure 2:**
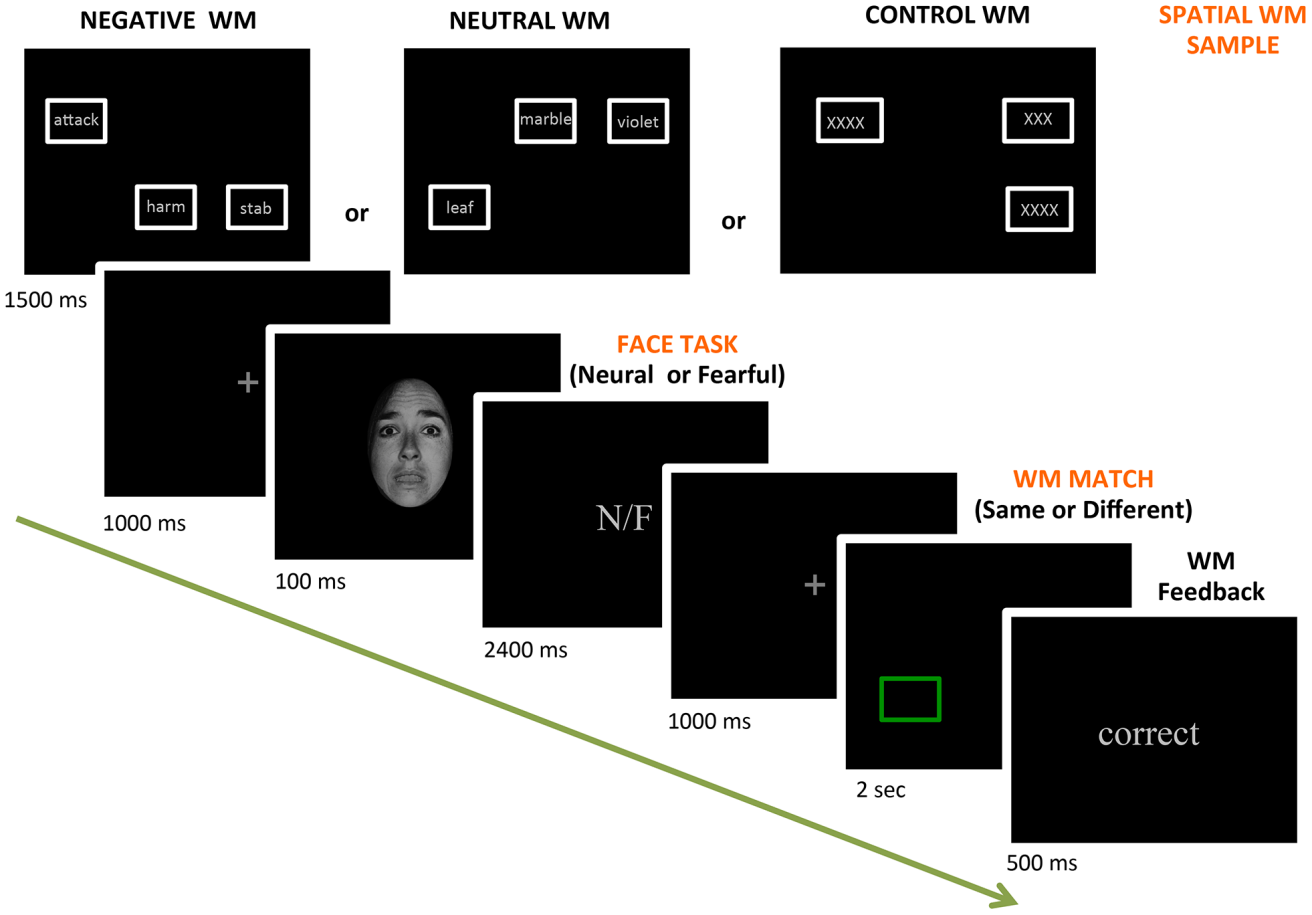
Experimental design. Three different types of spatial WM trials were presented in a blocked structure. Participants were asked to remember the location of white boxes presented at the beginning of trials and indicate whether the location of the green box presented later was the “same” (match) or “different” (non-match) from the previous locations. During the WM delay period, participants were required to make a “neutral” or “fearful” decision as quickly as possible. Stimuli are not drawn to scale.

Participants completed the dual-task paradigm, which involved a total of 240 trials. Each trial involved both a spatial location working memory (WM) task and a forced two-choice perceptual decision task (neutral vs. fearful). There were three different conditions of spatial WM trials: negative (NEG), neutral (NEU), and control (CON) trials, which were presented in a blocked fashion to minimize potential additional cognitive load due to task switches. A total of 15 WM blocks (3 WM types×5 blocks) were presented and each WM block contained 16 trials (4 identities ×4 emotional intensity levels). For each individual, the order of the blocks and the trial sequence within each block were fully randomized. The dual task trials began by displaying three white rectangle boxes on the computer screen (representing the WM sample display; see Figure 2 for examples). The locations of the boxes were randomly selected among 6 possible positions (top/bottom × left/middle/right) for each display, and the rectangle boxes were presented for 1.5 sec. For the NEG WM trials, the boxes contained negative words; for the NEU WM trials, they contained neutral words; and for the CON WM trials, they contained only the letter X, repeated. The word stimuli for NEG and NEU trials were randomly chosen from the word sets (3 words for each trials). The participants were to remember the location of the boxes on the screen (thus requiring spatial location WM), regardless of the words that appeared within. For each trial, an affective perceptual decision task was completed during the WM delay period. After the boxes (and words) disappeared, a 1-sec fixation cross was presented, followed by a 100-ms face stimulus. The brief stimulus presentation was employed in our experimental paradigm to eliminate or minimize the occurrence of deliberate eye saccades [47], as similarly done in previous studies [8], [17]. Participants were instructed to immediately indicate whether the face was neutral or fearful by a left-hand button press (number 1 and 2 on a keyboard). Participants were then instructed to maintain fixation at the center of a screen. Brief presentations of faces were employed to preclude deliberate saccadic eye movements. Participants were encouraged to make a decision as fast as possible and were given a total of 2.5 sec response window. After the face stimuli disappeared, another 1-sec fixation cross was presented, followed by a 2-sec spatial WM recognition test display. The WM test display contained one green box, the location of which was randomly chosen (50% match trials and 50% non-match trials). Participants were asked to indicate whether the spatial location of the test box was the same (match) or different (non-match) from any of the boxes presented during the previous WM sample display by a right-hand button press (number 9 or 0 on a keyboard). After a button press, a feedback screen (correct, incorrect, or miss) was presented for 500 ms. Every trial was separated by an inter-trial-interval of a 1.5 sec fixation cross. Before the main paradigm, participants completed a practice run containing 24 dual task trials in order to become familiar with the task. The schedule of stimulus presentation and behavioral data acquisition were controlled by Presentation software (Neurobehavioral System, CA).

### Psychometric Curve Fitting

To assess the effect of the working memory function on affective perception of facial expressions, we characterized participants' perceptual decision task performance via psychometric curves that related the proportion of “fearful” responses to the emotional intensity of the gradually morphed faces. In doing so, we utilized a psychometric curve fitting approach that has been successfully employed in previous studies of perceptual decision-making with emotional faces [8], [17]. Following the previous studies, psychometric curves were fitted by using the Naka-Rushton contrast response model [48], [49] with an OLS (Ordinary Least Square) criterion.
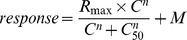



Here, *response* represents the proportion of “fearful responses” (indicating the face appears fearful), *C* is the graded emotional intensity level of the face (contrast: 20%∼80%), *C_50_* is the intensity at which response is half-maximal (also called “threshold” or “point of subjective equality: PSE”), *n* is the exponent that determines the slope of the response function, *R_max_* is the asymptote of the response function, and *M* is the response at the lowest stimulus intensity. The *R_max_* parameter was constrained to be equal or less than 1 and the *M* parameter was constrained to be equal or larger than 0. For each individual data, we fitted psychometric curves separately for each type of WM trial (NEG, NEU, CON). Curve fitting was performed with GraphPad Prism software (GraphPad Software, CA).

**Figure 3:**
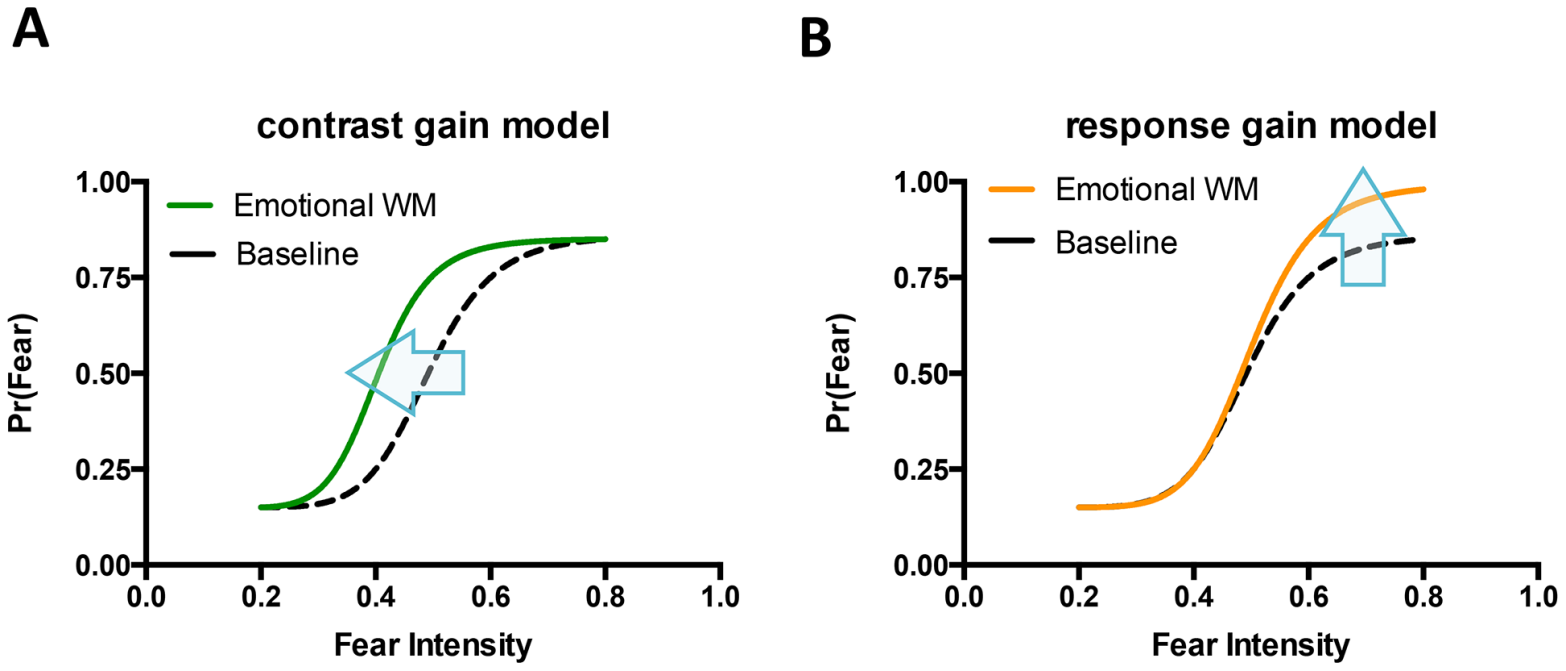
Contrast gain model and response gain model in the context of our experiment. **A**. Contrast gain model. A leftward shift of the psychometric curve (see arrow) would constitute evidence for decreased perceptual threshold for fearful decisions for face stimuli in emotional WM trials (i.e., participants having a lower emotional intensity threshold for deciding a face is fearful). **B**. Response gain model. A upward shift of the psychometric curve proportional to stimulus intensity would constitute evidence for enhanced response for fearful decisions for face stimuli in emotional WM trials (i.e., participants have the same threshold for deciding a face is fearful, but after reaching that threshold, being more likely to decide a face is fearful). The x-axis represents emotional intensity of face stimuli and the y-axis represents probability of fearful responses.

Based on the previous studies of affective perception [8], [17], we hypothesized that our WM manipulation would bias affective perception of facial expressions (neutral ∼ fearful) in a way consistent with either the contrast gain model and/or the response gain model. These two models have been used previously to explain the effect of attention on visual perception [38]–[41] (Figure 3). In general, the contrast gain model predicts that response changes in perception occur when the intensity of the stimulus is intermediate (consistent with a horizontal shift of psychometric curve; *C_50_* parameter shift). In our experiment, the contrast gain model would predict a decreased fear decision threshold (*C_50_* parameter), resulting in greater fear decisions at the intermediate levels of emotional intensity (40% or 60% fearful faces) by WM manipulation. On the other hand, the response gain model predicts that increases in response are proportional to the intensity of the stimulus and thus become evident at the high levels (consistent with a vertical shift of psychometric curve in the high levels; *R_max_* parameter shift). In our experiment, the response gain model would predict an increase in fear decisions at the high levels of emotional intensity (60% and 80% fearful faces). Previous studies [8], [17] that manipulated affective valence of the target stimulus or the background context have reported results consistent with the contrast gain model. However, these studies did not explore the influence of working memory manipulation on affective perception. Thus, in our experiment, the cognitive-affective working memory influence could occur in several different ways – e.g., a horizontal shift of psychometric curve in the intermediate level of emotional intensity (contrast gain model), or a vertical shift of psychometric curve in the high level of emotional intensity (response gain model).

## Results

### Working Memory Task

We compared the accuracies of spatial WM trials across the three experimental conditions. Note that the content of words presented in the WM task was task-irrelevant. The mean accuracies for NEG, NEU, and CON WM trials were 89.6% (*SE* = 1.1), 89.6% (*SE* = 1.2) and 98.1% (*SE* = 0.3). A repeated measure ANOVA on the WM task accuracy revealed a significant effect of WM type, *F*(2,74) = 33.09, *p*<.001, *η^2^* = .47. Subsequent planned post-hoc tests showed that participant's WM performance was significantly worse for NEG and NEU WM trials compared to CON WM trials, *t*(37) = −5.72, *p*<.001; *t*(37) = −6.37, *p*<.001. However, there was no significant difference between NEG and NEU WM trials in post-hoc tests, *p* = .98. This suggests that the task-irrelevant word stimuli, regardless of emotional content, had a disruptive effect on WM performance.

### Perceptual Decision Task

**Figure 4:**
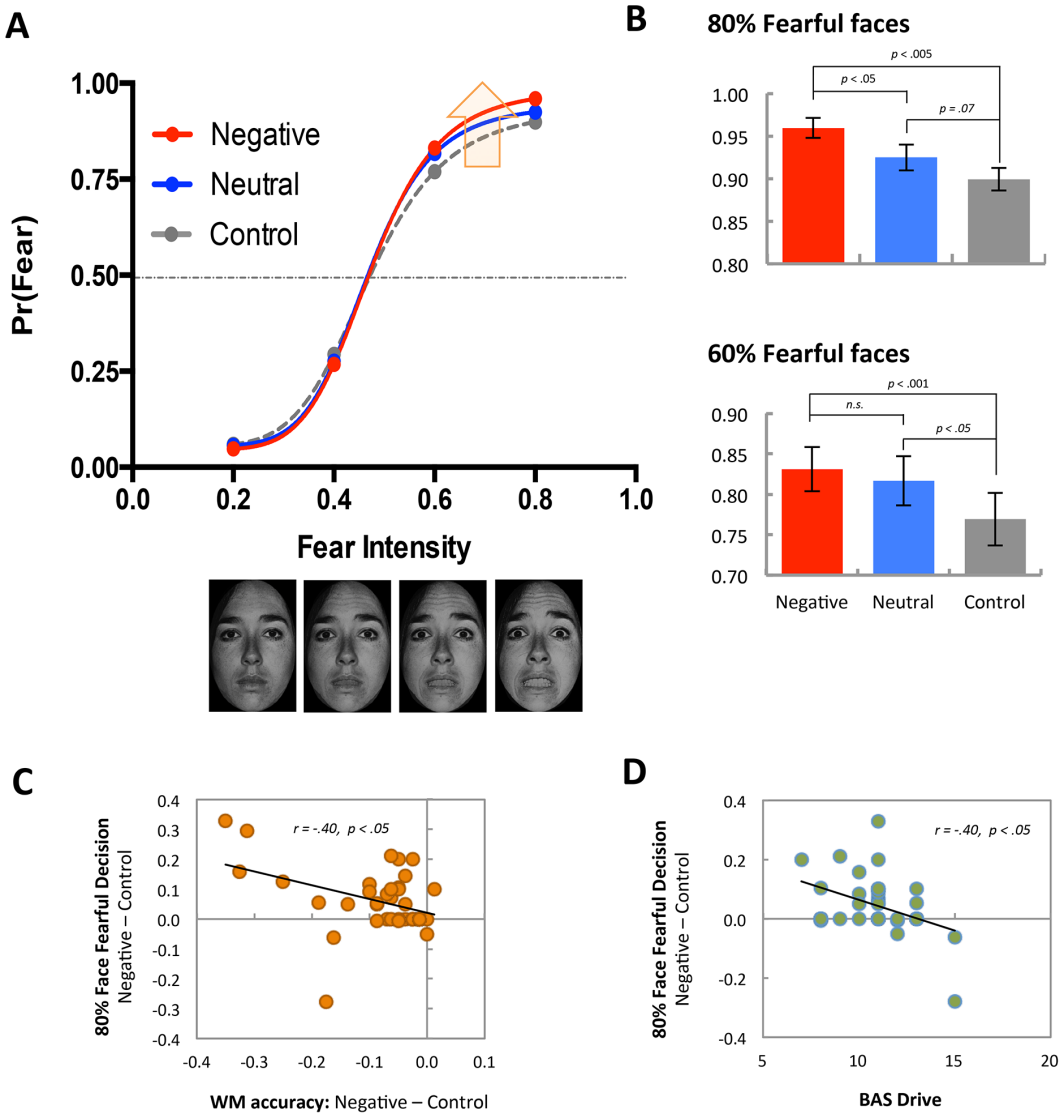
Behavioral findings. **A**. Average probability of fearful responses as a function of emotional intensity (20% to 80% fearful as shown in sample images) and WM condition. Red (negative WM), blue (neutral WM), and gray (control condition) lines represent the psychometric curves fitted by using the Naka-Rushton response function. **B**. Fearful decisions of 80% (top) and 60% (bottom) fearful face trials. Error bars denote the standard error of the mean. **C**. Scatter plot of the relationship between WM accuracy differences (NEG – CON) and 80% face fearful decision differences (NEG – CON). **D**. Scatter plot of the relationship between BAS Drive scale scores and 80% face fearful decision differences (NEG – CON). Solid line represents a linear fit.

To guarantee WM cognitive resource recruitment in our dual task condition, we excluded the trials in which participants did not answer correctly on the WM task. We then computed the proportion of “fearful” decisions for each type of WM condition. Average data for the perceptual decision task are shown in Table 1 and Figure 4A. To examine how task-irrelevant WM distractors (neutral and negative words) influence perceptual decision-making on emotional facial expression, we employed two different approaches. First, to quantitatively assess how cognitive-affective WM manipulation biased affective perception, we characterized observed responses via a fitting to the Naka-Rushton response function [8], [48], [49] for each individual's data (see Table 2 and Figure 4A). As stated earlier, we hypothesized that task-irrelevant negative word stimuli would influence perceptual decision making on facial expression (neutral ∼ fearful). That is, this influence could occur in several different ways – a horizontal shift of psychometric curve in the intermediate level of emotional intensity (contrast gain model) or a vertical shift of psychometric curve in the high level of emotional intensity (response gain model). The horizontal and vertical shifts of psychometric curves by WM type were tested by comparing the estimated *C_50_* (the perceptual decision threshold; PSE) and *R_max_* (the asymptote of responses) parameters of Naka-Rushton response model fits, respectively. While a repeated measures ANOVA on *C_50_* parameter did not show a significant effect, *F*(2,74) = .45, *n.s.*, *η^2^* = . 01, a repeated measure ANOVA on *R_max_* parameter showed a significant effect of WM type, *F*(2,74) = 3.25, *p*<.05, *η^2^* = .08. Subsequent planned post-hoc tests showed that the *R_max_* parameter of NEG WM trials was significantly higher than *R_max_* parameters of NEU and CON WM trials, *t*(37) = 2.74, *p*<.01; *t*(37) = 2.11, *p*<.05. No significant difference was found in the estimated *n* and *M* parameters, *F*(2,74) = 0.43, *n.s.*; *F*(2,74) = 0.39, *n.s.* These results indicate that the task-irrelevant negative word content presented during the WM sample period had a subsequent influence on perceptual decision-making, characterized by increases in fear decisions at the high intensity levels of emotional expression. This finding is consistent with the response gain model.

**Table 1:** Means and standard errors of fearful decisions.

	Intensity Level of Morphed Faces			
Working Memory Type	*20% fearful*	*40% fearful*	*60% fearful*	*80% fearful*
Negative words	.048 (.011)	.268 (.029)	.831 (.028)	.959 (.011)
Neutral words	.057 (.010)	.276 (.032)	.817 (.030)	.925 (.015)
Control	.060 (.011)	.294 (.029)	.769 (.033)	.899 (.071)

**Table 2:** Means and standard errors of psychometric curve fit parameters.

Working Memory Type	*C_50_*	*R_max_*	*n*	*M*
Negative words	.448 (.011)	.983 (.006)	15.250 (2.393)	.003 (.002)
Neutral words	.446 (.012)	.946 (.013)	20.403 (2.571)	.005 (.003)
Control	.457 (.014)	.959 (.007)	16.333 (1.920)	.004 (.003)

Next, to confirm the robustness of findings we performed a 3 (WM type) by 4 (Emotion Intensity) repeated measure ANOVA on behavioral data of proportions of fearful decisions, which does not require any assumption for the shape of psychometric curves. The ANOVA result on fearful responses revealed a significant WM type × Emotion Intensity interaction effect, *F*(6,222) = 4.52, *p*<.001, partial *η^2^* = .11, and a significant main effect of Emotion Intensity, *F*(3,111) = 19.84, *p*<.001, partial *η^2^* = .93. There was no significant main effect of WM type, *F*(2,74) = 2.34, *n.s.* To clarify this interaction effect, we performed simple effect analyses in which the effect of WM type was tested for each level of Emotion Intensity. Consistent with the previous psychometric fit results that supported the response gain model's prediction (i.e., increases of fear perception in the high level of emotional intensity), we observed significant effects of WM type in 60% and 80% high Emotion Intensity levels, *F*(2,74) = 5.34, *p*<.01, *η^2^* = .13; *F*(2,74) = 8.36, *p*<.001, *η^2^* = .18, but not in 20% and 40% low Emotion Intensity levels, *F*(2,74) = 0.87, *n.s.*; *F*(2,74) = 0.82, *n.s.* Again, the response gain model's prediction was supported. As shown in Figure 4B, in both 60% and 80% levels, participants made fearful perceptual decisions more frequently in NEG WM trials compared to CON WM trials, *t*(37) = 3.41, *p*<.001; *t*(37) = 3.50, *p*<.005. Also, participants made more frequent fearful decision in NEU WM trials compared to CON WM trials in 60% intensity level, *t*(37) = 2.11, *p*<.01, as well as in NEG WM trials compared to NEU WM trials in 80% intensity level. *t*(37) = 2.67, *p*<.01.

### Correlation Analysis

To investigate the relationship between WM task performance and biased affective perception, we performed a correlational analysis. In this analysis, we used differential scores to index WM performance difference (NEG WM – CON WM accuracy) and perceptual decision bias (fearful decision in NEG WM trials – fearful decision in CON WM trials) induced by task-irrelevant negative words. As shown in Figure 4C, at the maximum 80% fear intensity level, the perceptual decision bias (increased fearful decisions induced by NEG WM) showed a significant negative correlation with WM performance differences between NEG and CON WM trials, *r*(36) = −.40, *p*<.05. This finding suggests that the level of cognitive demand experienced by a participant due to the task-irrelevant emotional distractors might underlie the impact these distractors had on emotional face perception. To further explore whether this perceptual decision bias correlates with individual differences, we performed an additional exploratory correlational analysis with STAI and BIS/BAS self-report scales. Similar to the previous study [8], the perceptual decision bias showed a negative correlation with the BAS Drive scale, *r*(31) = −.40, *p*<.05 (Figure 4D). This finding suggests that the strength of the activation or reward system may reduce the impact of task-irrelevant distractors on subsequent emotional face perception. A similar correlation with NEU WM – CON WM accuracy did not reveal significant findings.

## Discussion

Our study clearly demonstrates that facial emotion perception is influenced by both the cognitive-affective state of a perceiver, as well the emotional expressions of the perceived face. When either neutral or negative emotional words were presented as task-irrelevant working-memory distractors in the dual task paradigm, participants more frequently reported fearful face perception in the 60% and 80% fearful face conditions. This produced a vertical shift of the psychometric curve at the higher emotional intensity levels of morphed faces, consistent with the response gain model [38].

There are several important issues necessary to clarify before an interpretation of our findings. Most importantly, it should be noted that we found the significant differences only in a certain range of emotional intensity (60% and 80%) and not in all emotional intensity levels presented in this study. This therefore cannot be explained by a *general* performance decrease due to the additional working memory task. Similarly, affective priming (by pre-exposure of emotional words) or a congruency effect of word stimuli and faces, cannot fully explain our results. If this were the case, we would expect a linearly increasing effect only for negative WM trials (i.e., an interaction between emotional intensity and NEG/NEU WM conditions). Instead, we observed similar effects (with different magnitude) with neutral words as well as negative words. Finally, while our main interest was the influence of WM distractors on affective face perception, the presentation of emotional faces could have influenced subsequent WM performance (e.g., higher emotional intensity or higher ambiguity of faces would differentially modulate WM retrieval). However, it is unlikely that this fully explains our findings, as the emotional intensity level of the faces did not have a significant impact on WM performance during the CON WM conditions (*p* = .36).

Interestingly, our finding that working memory distractors only impacted face perception at the 60% and 80% emotional intensity levels conflicts with previous findings, which showed an overall accuracy *decrease* for an emotion identification task during the relatively difficult 2-back working memory task [50]. One potential reason this previous study did not demonstrate such cognitive-emotional interactions may be because it did not include the full range of emotional intensities [15]. Despite identical physical features of faces in the current study, task-irrelevant negative word stimuli presented during the WM task only influenced the frequency of “fearful” responses at the high levels of facial emotional intensity. As a consequence, the dynamic range of fearful responses (maximum response rate – minimum response rate) was increased in the negative WM condition. This pattern is consistent with the response gain model, which predicts that an increase in fearful perception occurs mostly when the emotional intensity of stimuli is high.

In this study, affective perception of facial expressions was not fully independent from ongoing executive function manipulated by task irrelevant WM distractors. Several previous studies [51], [52] have shown that facial expression recognition performance can be significantly benefitted by successful inhibition or attentional control of task-irrelevant distractors, although these studies did not directly manipulate WM distractors. Thus, it is plausible that explicit attentional control mechanisms that consume cognitive resources were systematically affected by emotional or non-emotional WM distractors in our task. Our results suggest that affective perception may require processing resources that are partly shared with concurrent working memory tasks. When fewer resources were available for the face decision task, due to the previously presented negative emotional distractors, participants showed a tendency to perceive ambiguous facial expressions as more “fearful” at high emotion intensities (60∼80% intensities above the perceptual decision threshold).

What are the social implications of these findings? Decoding of expressed facial emotion plays a critical role in social interaction. The systematic affective appraisal bias that would subsequently evoke biologically programmed fear response mechanisms (i.e., fight-or-flight) may have ecologically adaptive value for increasing one's survival odds in a situation with few available cognitive resources. For example, when given a difficult task, such as caring for young or going out hunting, one would be more sensitive to faces of high emotional intensity, perhaps increasing the speed at which one could respond to the situation – while not sacrificing the accuracy of perception of more neutral faces. This would therefore also minimize energy expenditure due to false alarms. In more modern terms, individuals with high cognitive demands in the workplace in addition to intrinsic personal or emotional distractors may be more sensitive to high-intensity emotional faces. In clinical populations who suffer from compromised processing resources due to greater cognitive or emotional demands, this effect may be further exaggerated.

In this study, we didn't observe any systematic differences in one's perceptual decision threshold (*C_50_*; PSE) by the type of WM manipulation. In other words, our results imply that there was no change in the categorical decision boundary between neural and fearful expression. It is not immediately apparent why our WM manipulation revealed a vertical shift that is consistent with the response gain model, while the previous studies [8], [17] showed a horizontal shift that is consistent with the contrast gain model. Future, larger replication studies are required to rule out type I and/or type II errors that might exist in these studies to make a conclusion about this discrepancy. Yet, there were two distinctive features of our current design compared to the previous experiments [8], [17]. First, our study included the WM task as well as the perceptual decision task of facial expressions. Second, there was no discriminative or additional visual information provided during the face task. In Lim and Pessoa's study (2008), affective significance of face stimuli (manipulated by fear conditioning) was signaled by the color of faces (red or blue; counterbalanced across participants). In Lee and colleagues' experiment (2012), emotional context conditions were manipulated by the surrounding visual images (simultaneously presented visual IAPS images). Emotional face processing engages multiple levels of processing in a widely distributed network of brain areas [29], [53], [54]. Affective or cognitive context modulation of face processing can occur at an early visual processing stage involved in face encoding, at a late stage involved in decision-making and interpretation, or a combination of both [4]. In the domain of visual processing, the contrast gain model is often believed to operate at the earlier stage, while the response gain model is often suggested to reflect changes occurring at the later stage such as decision-making by recruiting different brain mechanisms [40]. Whether available cognitive resources modulate the “later” stages of affective perception that may be more directly linked to elaborate decision-making, awaits further investigation.

Individual differences in how one processes affective information indexed by the BIS/BAS scales are known to be one of modulatory factors of affective perception of facial expressions [8], [55], [56]. In the current study, we found a significant negative relationship between affective perception bias and the behavioral activation system (BAS) Drive scale. The participants with low BAS drive scores tended to more frequently make fearful decisions for the 80% fearful faces in the NEG WM condition compared to the NEU WM condition. Traditionally, the BAS has been related to approach motivation such as reward processing or positive affect, while the behavioral inhibition system (BIS) has been related to avoidance motivation or negative affect [43]. However, the BAS trait is often associated with the behavioral and neural processing of negative emotional expressions. For example, the BAS drive scores related to increased behavioral tendencies to display angry reactions to failure [57], [58] as well as increased fMRI responses to angry facial expressions in the amygdala, a region critically involved in the affective perception [24]. In our study, fearful decisions showed a negative correlation, not a positive correlation with BAS drive scale. Although fearful and angry expressions are often grouped to “fear-related” category, they convey very different information to the perceiver; fearful facial faces provides information about the increased probability of an environmental threat, whereas angry facial expressions embody a certain and direct threat from the individual [59]. In addition, anger has often been theorized as an “approach” drive, while fear may be more related to “avoidance” drives [24], [59], [60]. This could translate into those with high approach drives to respond very differently to anger as compared to fearful stimuli. However, further research is required to determine the exact underlying mechanisms between behavioral activation and inhibitions systems and affective perception of facial expression.

Overall, the results of this study provide support that cognitive-affective interaction matters for the affective perception of facial expression. One strength of the study was the rigorous, highly-specific empirical task used to assess the effect of cognitive-affective interaction on facial perception. Our study clearly demonstrates that facial emotion perception is influenced by both the cognitive state of the perceiver as well the emotional expressions of the perceived face. This could indicate that if working memory is over-taxed (i.e., by increased life demands, brain damage, mental illness, or stress), emotional judgments could be compromised. Thus, results could have implications for understanding cognitive-emotional interactions in the case of neurologic and psychiatric disorders. However, future research is needed to explore how the working memory function influences affective perception of facial expressions a variety of clinical populations.
